# Recent developments of the reconstruction in magnetic particle imaging

**DOI:** 10.1186/s42492-022-00120-5

**Published:** 2022-10-01

**Authors:** Lin Yin, Wei Li, Yang Du, Kun Wang, Zhenyu Liu, Hui Hui, Jie Tian

**Affiliations:** 1 CAS Key Laboratory of Molecular Imaging, the State Key Laboratory of Management and Control for Complex Systems, Institute of Automation, Chinese Academy of Sciences, Beijing, 100190 China; 2 Beijing Key Laboratory of Molecular Imaging, Beijing, 100190 China; 3 University of Chinese Academy of Sciences, Beijing, 100049 China; 4 Medical Imaging Center, the First Affiliated Hospital, Jinan University, Guangdong, 510632 China; 5 Beijing Advanced Innovation Center for Big Data-Based Precision Medicine, School of Medicine, Beihang University, Beijing, 100083 China

**Keywords:** Magnetic particle imaging, Image reconstruction, System matrix, X-space

## Abstract

Magnetic particle imaging (MPI) is an emerging molecular imaging technique with high sensitivity and temporal-spatial resolution. Image reconstruction is an important research topic in MPI, which converts an induced voltage signal into the image of superparamagnetic iron oxide particles concentration distribution. MPI reconstruction primarily involves system matrix- and x-space-based methods. In this review, we provide a detailed overview of the research status and future research trends of these two methods. In addition, we review the application of deep learning methods in MPI reconstruction and the current open sources of MPI. Finally, research opinions on MPI reconstruction are presented. We hope this review promotes the use of MPI in clinical applications.

## Introduction

**Fig. 1:**
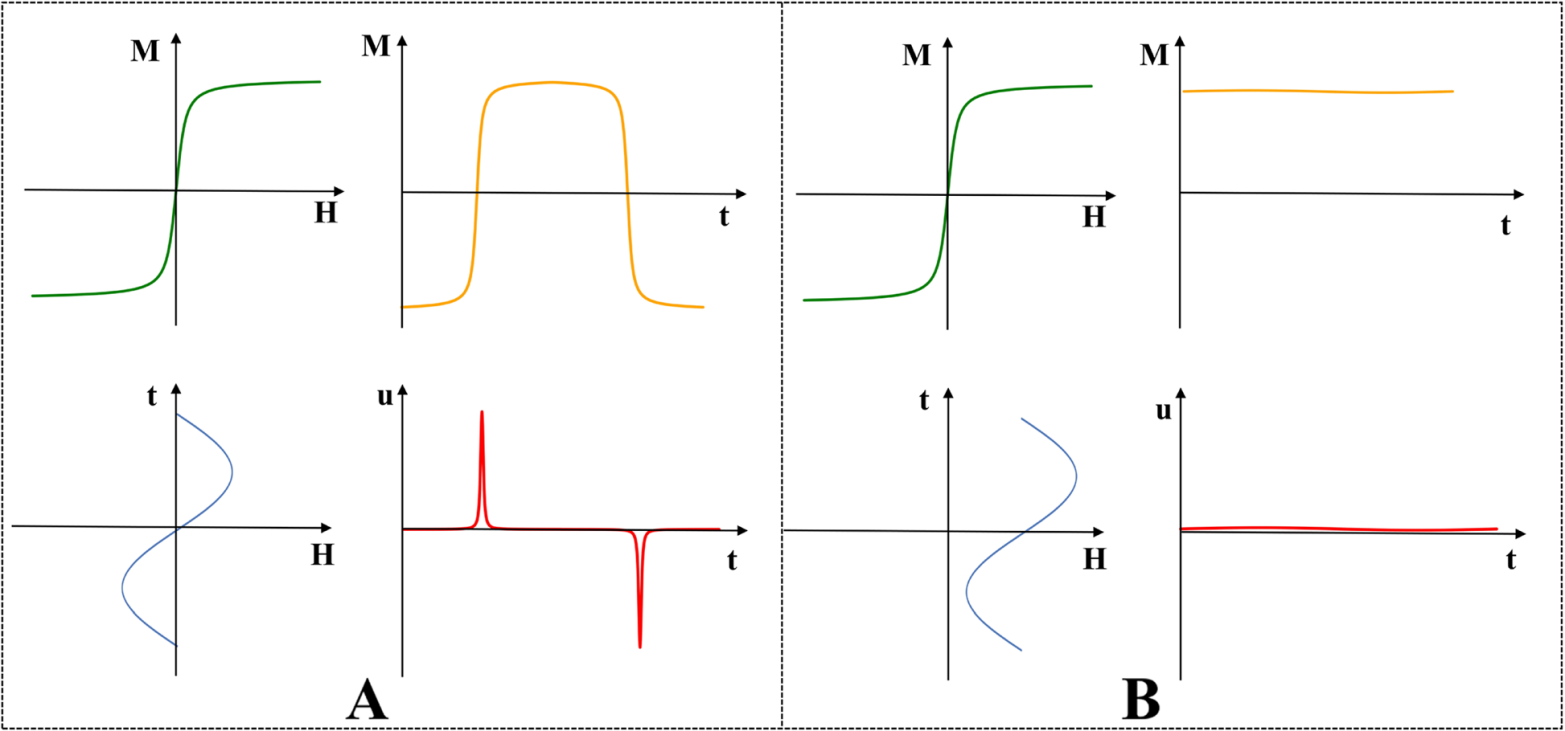
Magnetization response of SPIOs. **A** SPIOs are excited with a sinusoidal magnetic field. **B** SPIOs enter the magnetized saturation region

Magnetic particle imaging (MPI) is a novel molecular imaging modality that can image superparamagnetic iron oxide particles (SPIOs) with high temporal-spatial resolution and sensitivity in a noninvasive manner [1–4]. In 2005,Gleich and Weizenecker [1] first proposed the physical principles and wide application prospects of MPI. The imaging principle of MPI is related to the nonlinear magnetization response of SPIOs in a magnetic field. First, a gradient field generated by a permanent magnet or energized coil is used to create a field-free region (FFR). Subsequently, by superimposing an oscillating drive field, the FFR moves through the field of view (FOV) [5, 6]. The excitation field influences SPIOs near the FFR to undergo a nonlinear magnetization response to generate a voltage signal in the receiving coils (Fig. 1 A). When SPIOs enter the magnetic-field saturation region, SPIOs are not magnetized to produce voltage signals (Fig. 1 B). The concentration distribution of the SPIOs can be further obtained by analyzing the reconstruction algorithm from the signal to the image [7, 8].

Compared with current powerful imaging techniques, MPI has significant advantages. Computed tomography (CT) and magnetic resonance imaging (MRI) can achieve a sub-millimeter spatial resolution, but their sensitivity is limited, resulting in poor specificity at the molecular level [4]. Positron emission tomography (PET) and single-photon emission computed tomography (SPECT) have high sensitivity but a spatial resolution of approximately 3 mm. In addition, these applications are limited by the short half-life of radioactive tracers in cell tracking or other several research fields that require long observations [9]. Optical imaging technology can achieve a high sensitivity and spatial-resolution imaging, but its imaging depth is limited to 2-3 cm [10, 11]. MPI is expected to overcome the limitations of molecular imaging technology in terms of imaging depth, sensitivity, resolution, and radiation, thereby becoming a new trend in the development of high-end medical imaging that represents the international academic frontier of the development of modern medical imaging [4, 12–14]. Table 1 presents quantitative comparisons between different imaging modalities.

**Table 1:** Quantitative comparisons of different imaging modalities

**Property**	**CT**	**MRI**	**PET/SPECT**	**Optical imaging**	**MPI**
Spatial resolution	< 1 mm	< 1 mm	3 mm	< 1 mm	< 1 mm
Temporal resolution	1 s	1 s-1 h	1 min	< 0.1 s	< 0.1 s
Sensitivity	Low	Low	High	High	High
Depth	High	High	High	2-3 cm	High
Radioactivity	Yes	No	Yes	No	No

As a new molecular imaging technology, MPI plays an increasingly important role in many preclinical biomedical studies. MPI has been proven to achieve a highly sensitive detection of approximately 250 cells in vivo in cell tracking studies [15]. MPI has provided the highest imaging sensitivity in multimodality dynamic observations of gliomas [16, 17]. MPI can also detect and visualize the homing of breast tumor cells with high sensitivity [18]. MPI has also been successfully applied in cardiovascular and cerebrovascular imaging [19, 20] and neuroimaging [21]. These advances fully demonstrate the imaging advantages of the high sensitivity and specificity of MPI, demonstrating the great potential and value of this new molecular imaging technology.

**Fig. 2:**
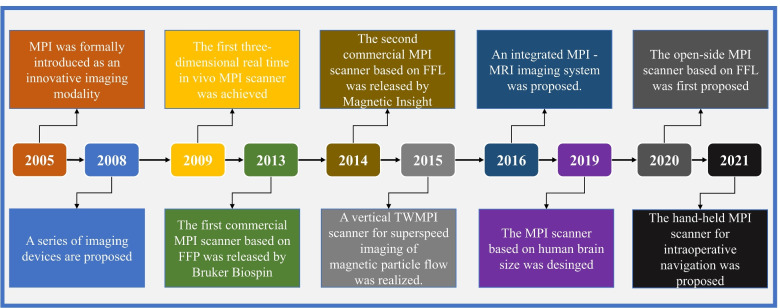
Timeline of MPI development

In recent years, the imaging theory and instruments for MPI have been continuously improved and developed. In 2008, instead of moving the object mechanically in a horizontal direction, Gleich et al. [22] achieved fast two-dimensional (2D) imaging by superposing a horizontal drive field. In the same year, they proposed a field-free line (FFL), which is an effective encoding scheme for MPI. The results of the simulations demonstrated an obvious improvement in image quality compared with the existing field-free point (FFP) [23–26]. Subsequently, novel single-sided MPI equipment was proposed by Sattel et al. [27], which overcame the limitations of specimen size. In 2009, they achieved the first three-dimensional (3D) real-time in vivo MPI [2]. In 2013, the world’s first commercial MPI scanner (based on FFP) was released by Bruker Biospin. In 2014, the Magnetic Insight company from the United States unveiled the world’s second commercial MPI scanner (based on FFL) [4]. In 2015, a superspeed traveling wave MPI scanner was proposed to realize superspeed imaging of magnetic particle flows [28]. In 2016, Franke et al. [29] designed a hybrid system that combined MRI and MPI. In 2019, Gräser et al. [30] designed an MPI scanner with the size of the human brain. In 2020, scientists from Turkey proposed an open-sided MPI scanner system based on the FFL scheme [31]. In 2021, a novel handheld MPI scanner was designed for the intraoperative imaging of breast nodules [32]. Figure 2 shows the development timeline of MPI.

With the continuous development of MPI scanners, abundant research on MPI reconstruction has been conducted to enhance the imaging performance of the instruments. MPI reconstruction algorithms are primarily of two types of methods: system matrix (SM) and x-space. SM-based MPI reconstruction is an important field [33–35]. The acquisition of SMs in SM-based MPI reconstructions is an important research topic. Currently, three methods have been reported for obtaining SMs: measurement-based SM, sparse recovery SM, and model-based SM. Owing to the complex magnetic environment and magnetization behavior of SPIOs, the development of an accurate physical model remains a challenging problem. Therefore, the first reported, and the most accurate method, is measurement-based SM acquisition, which involves moving a delta sample with a robot at all positions of the entire FOV [1, 36]. Despite its high accuracy, the measurement process is time consuming. In addition, the measured SM requires a lot of memory, which leads to inefficient reconstruction. Therefore, sparse recovery methods and model-based SM acquisition have been proposed [37, 38]. In addition to the SM acquisition method, a reconstruction strategy based on SM is an important research topic. Further details regarding this are covered later in this review.

X-space methods are another important component of MPI reconstruction [39–42]. This study reviews the basic theory and improved algorithms developed in recent years. In addition, with the current development of artificial intelligence (AI), strategies based on deep learning networks have also shined in the field of MPI reconstruction. We review the relevant research in detail. Furthermore, there is an increasing number of MPI research groups around the world, as well as many open-source data, programs, and software platforms, which are also discussed in this review. Finally, the conclusions and outlook of MPI are presented.

## SM-based MPI reconstruction

The objective of MPI reconstruction is to transform the induced voltage signal into a spatial concentration distribution of the SPIOs. A SM is a mapping of the two items, considering the complex magnetic field and SPIOs properties [7, 36, 43]. The linear mapping between the induced voltage signal $u_k$ ( Fourier coefficients of the time-domain signal *u*(*t*)) and SPIO concentration $c(\mathbf {r})$ can be described as follows:

1 $$\begin{aligned} u_k = \int _\Omega s_k(\mathbf {r})c(\mathbf {r})d^3\mathbf {r}, ~~k=1,\dotsc , K \end{aligned}$$

where $\mathbf {r}$ represents the spatial position, and $s_k(\mathbf {r})$ denotes the system function. K denotes the total number of frequency components. By sampling all N positions of the FOV, we obtain the following linear relationship:

2 $$\begin{aligned} \mathbf {u}=\mathbf {S}\mathbf {c} \end{aligned}$$

where $\mathbf {S}\in \mathbb {C}^{K\times N}$ denotes the SM. $\mathbf {u} \in \mathbb {C}^{K\times 1}$ and $\mathbf {c} \in \mathbb {R}^{N\times 1}$ denote the voltage and SPIOs concentration vector, respectively.

SM-based reconstruction plays an important role in MPI reconstruction. This section describes the commonly used SM acquisition methods and reconstruction strategies based on SM.

### Acquiring the SM

#### Measurement-based methods

**Fig. 3:**
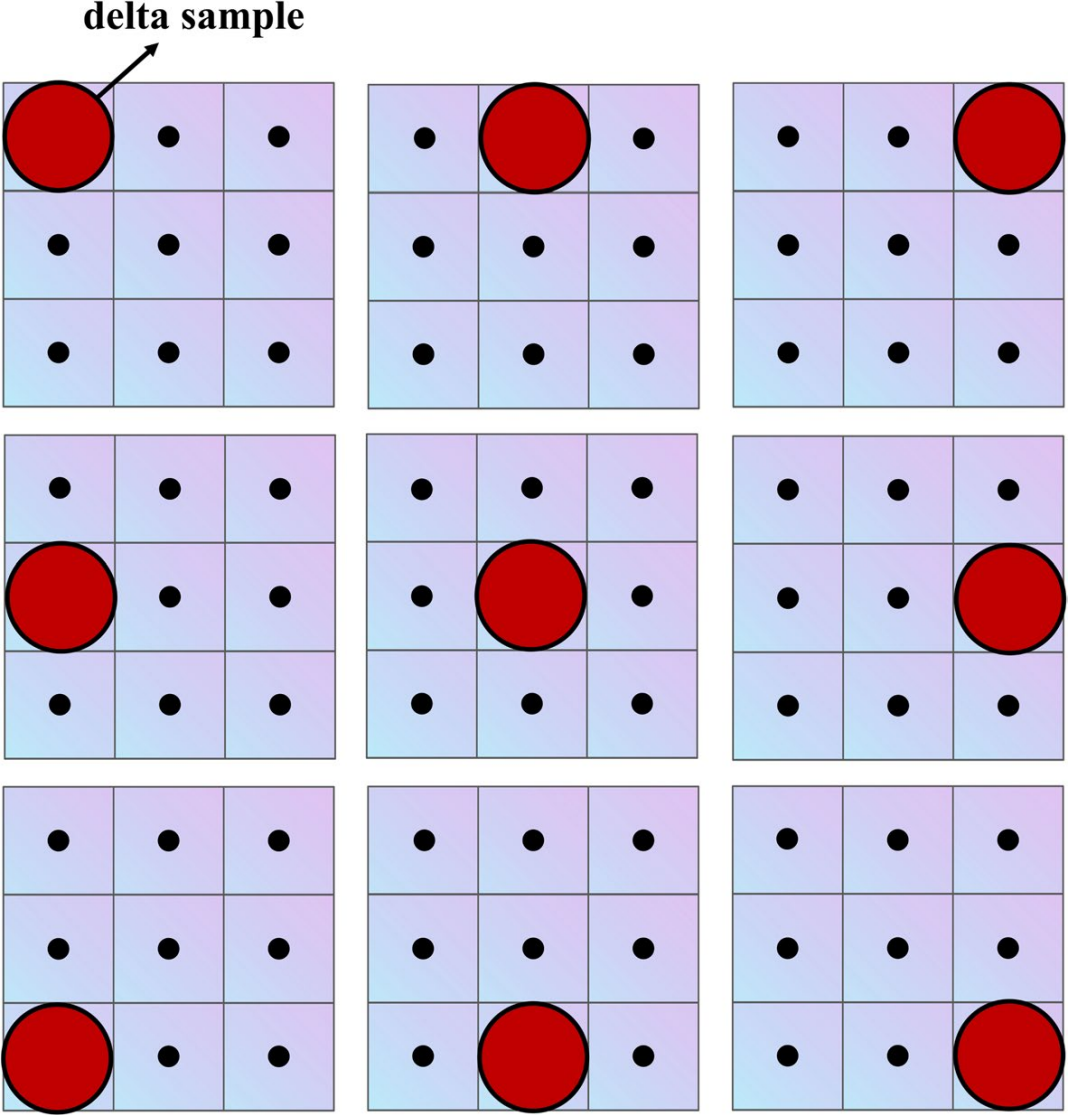
Principle of measurement-based SM method of MPI using a delta sample (taking a grid of 3$\times$3 as an example). A robot moves the delta sample to scan the location of each pixel

A tedious calibration procedure must be performed for the measurement-based SM acquisition method. Figure 3 shows the calibration procedure for the measurement-based method. It measures the induced voltage signal at all positions in the FOV with a delta sample to obtain the SM. Even a medium-sized image of 34$\times$28$\times$20 can require up to six hours to measure the SM [2, 37]. Therefore, a high-quality image requires a time-consuming and tedious calibration procedure. In addition, when the experimental conditions change, such as changes in particle species and magnetic field intensity, the SM must be recalibrated. In addition, cooling is required during calibration because the coils overheat after prolonged system operation [44].

##### Sparse recovery methods

Owing to the complexity and time requirements of the measurement-based SM acquisition method, many studies have proposed sparse recovery methods to obtain a complete SM using only a subset of all calibration scans. In 2013, Knopp and Weber [37] proposed using the sparsity of SMs based on certain basis transformations (such as the discrete Fourier transform, discrete cosine transform or discrete Chebyshev transform) for compressed sensing (CS) reconstruction to significantly reduce the number of calibration scans. Each row of the SM is handled separately. Taking the k-th row as an example, an inverse problem must be solved:

3 $$\begin{aligned} \underset{\mathbf {x}}{\arg \min } \ \ \Vert \mathbf {\Phi } \mathbf {x}\Vert _1 ~~~~\text {subj.to}~ \frac{1}{2}\Vert \mathbf {Px}-\mathbf {y}_k\Vert _2^2<\varepsilon ^2 \end{aligned}$$

where $\mathbf {\Phi }\in \mathbb {C}^{N\times N}$ denotes a basis transformation matrix, $\mathbf {x}\in \mathbb {C}^{N\times 1}$ denotes the optimization results, and $\mathbf {P}$ represents the undersampled matrix, which denotes the position index of the points sampled from the k-th row $\mathbf {s}_k$. $\mathbf {y}_k$ denotes the undersampled measurement: $\mathbf {y}_k=\mathbf {P}\mathbf {s}_k$. Several algorithms have been proposed to solve this inverse problem [37]. The sparse recovery method based on the standard CS can reduce the sampling rate by 10% [37].

**Fig. 4:**
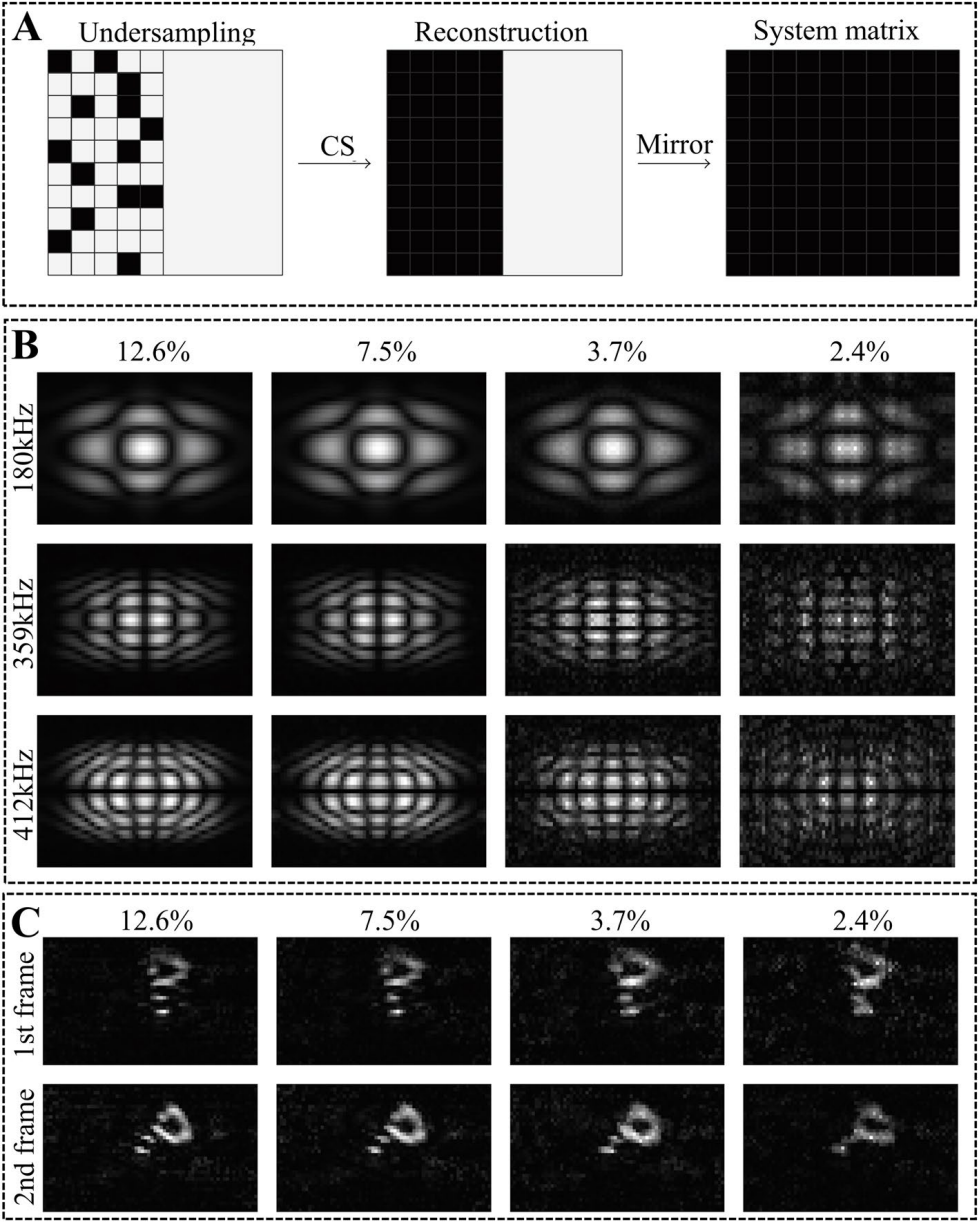
**A** The principle of the CS-symmetry method: the first half of the SM is recovered based on the CS method. Then, the complete SM is recovered based on mirror symmetry (alternatively, by changing the order of the two steps, symmetry-CS is permissible). **B** The SM components at different frequencies recovered with the symmetry-CS method at different sampling rates. **C** Reconstructed phantom results of the recovered SM at different sampling rates based on the symmetry-CS method. For more images see ref. [45]

Based on CS theory, many improved sparse recovery algorithms have also been extended. Weber and Knopp [45] proposed the use of SM symmetry to further compress SMs (Fig. 4). Grosser et al. [44] proposed the use of low-rank tensors to represent SMs, which allows SM recovery even when the sampling rate is reduced to 2%. Ilbey et al. [46] presented a framework for a coded calibration scene. Compared with the standard CS method, the image quality is significantly improved under the same signal-noise ratio (SNR) and sampling rate [46]. Sparse recovery algorithms can significantly reduce the calibration time and guarantee the quality of image reconstruction, which has attracted increasing attention in the field of MPI reconstruction.

##### Model-based methods

In addition to the measurement-based and sparse recovery methods, Knopp et al. [38] proposed a model-based method in 2010. They used the model of a signal chain to generate SMs. This approach consisted of three steps.

1) Signal encoding: the principle of the signal chain is used to model signal encoding. In MPI, the relationship between the time-domain induced voltage *u*(*t*) in the receiving coil and SPIO concentration $c(\mathbf {r})$ is as follows:

4 $$\begin{aligned} u(t)=-\mu _0 \int _\Omega \frac{\partial }{\partial t}\mathbf {M}(\mathbf {r},t)\cdot \mathbf {p}(\mathbf {r})c(\mathbf {r}) d\mathbf {r} \end{aligned}$$

where $\mu _0$ represents the permeability of a vacuum, and $\mathbf {M}(\mathbf {r},t)$ denotes the unit magnetization of the SPIOs. $\mathbf {p}(\mathbf {r})$ is the sensitivity profile of the recording coil. Because filtering is easier in the frequency domain, processing is generally performed in the frequency domain to increase the accuracy of the modeled-induced voltage:

5 $$\begin{aligned} u_k=\int _\Omega \left( a_k \int _{0}^{T} \frac{\partial }{\partial t}\mathbf {M}(\mathbf {r},t)\cdot \mathbf {p}(\mathbf {r}) e^{2\pi ikt/T}dt\right) c(\mathbf {r}) d\mathbf {r} \end{aligned}$$

where $a_k$ is the transfer function. Comparing Eqs. (1), (4), and (5), the model-based system function is obtained as follows:

6 $$\begin{aligned} s_k^{model}(\mathbf {r})=a_k\int _{0}^{T}\frac{\partial }{\partial t}\mathbf {M}(\mathbf {r},t)\cdot \mathbf {p}(\mathbf {r}) e^{2\pi ikt/T}dt \end{aligned}$$

2) Particle model: Langevin is the most commonly used model for describing the magnetization of SPIOs:

7 $$\begin{aligned} \mathbf {M}(\mathbf {r},t)=\mathbf {e_H}\int _{0}^{\infty }\rho (D)m\left( coth(\xi )-\frac{1}{\xi } \right) dD \end{aligned}$$

with

8 $$\begin{aligned} \xi =\frac{\mu _0m\Vert \mathbf {H}(\mathbf {r},t)\Vert _2}{k_BT} \end{aligned}$$

where $K_B$ and T are the Boltzmann constant and temperature, respectively. *m* denotes the magnetic moment, and $\mathbf {e_H}=\mathbf {H}(\mathbf {r},t)/\Vert \mathbf {H}(\mathbf {r},t)\Vert _2$ denotes the direction of the magnetic field strength. D represents the diameters of the SPIOs.

3) Transfer function: $a_k$ can be obtained by minimizing the following equation:

9 $$\begin{aligned} f(a_k)=\int _\Omega |s_k^{meas}(\mathbf {r})-a_k \hat{s}_k^{model}(\mathbf {r})|^2d\mathbf {r} \end{aligned}$$

where *k* denotes the frequency index, $s_k^{meas}$ denotes the measured system function, and $\hat{s}_k^{model}=s_k^{model}/a_k$. $a_k$ can be computed as follows.

10 $$\begin{aligned} a_k=\frac{\int _\Omega s_k^{meas}(\mathbf {r}) \overline{\hat{s}_k^{model}(\mathbf {r})}d\mathbf {r}}{\int _\Omega |\hat{s}_k^{model}(\mathbf {r})|^2d\mathbf {r}} \end{aligned}$$

In principle, a measured system function with a single position is sufficient for obtaining $a_k$. This approach demonstrates the feasibility of modeling instead of measuring SMs [38]. Subsequently, Knopp et al. [47] verified the accuracy of a model-based SM acquisition method on 2D data. Model-based SM acquisition methods can accurately model the system function based on a few calibration scans, and the accuracy can be increased by improving the underlying physical model.

### SM-based MPI reconstruction based on the regularization strategy

After obtaining the SM, the concentration distribution of the SPIOs can be obtained by solving Eq. (2) using a series of optimization algorithms. Owing to the ill-posedness of the inverse problem, a regularization strategy was introduced to improve the accuracy of image reconstruction. Tikhonov regularization is the most commonly used method and is widely used in MPI reconstruction owing to its fast and simple implementation [3]:

11 $$\begin{aligned} \underset{\mathbf {c}\ge 0}{\arg \min }\Vert \mathbf {W}^{1/2}(\mathbf {Sc-u})\Vert ^2_2+\lambda \Vert \mathbf {c}\Vert ^2_2 \end{aligned}$$

where $\lambda$ denotes the regularization parameter that penalizes the solution with a large Euclidean norm. $\mathbf {W}$ denotes the weighting matrix used to normalize the elements of the SM.

Both direct and iterative methods are used to solve the Tikhonov regularization. Examples of direct methods are Cholesky decomposition or singular value decomposition (SVD) [48, 49]. Direct methods have several advantages. For example, SVD can flexibly tune the regularization parameters with only a small amount of computational effort. However, the complete SM must typically be stored in memory while solving Eq. (11), which is unfeasible for a huge SM of 3D MPI.

Therefore, iterative methods have emerged with a high demand in MPI, leading to less memory and computational effort in several cases. Popular iterative methods include the conjugate gradient method [48] and Kaczmarz method [50, 51]. The Kaczmarz method is a row-action method that operates on rows independently. Convergence has been reported to occur within ten iterations of the Kaczmarz method [52]. Therefore, the state-of-the-art method for SM-based reconstruction is the Tikhonov regularization solved by the Kaczmarz method [53]. In recent years, many Kaczmarz-related Tikhonov regularization solutions have emerged in the field of MPI reconstruction [3, 50, 54].

Although Tikhonov regularization is simple and easy to implement, it does not use the inherent spatial neighborhood structure priori to improve the image quality. Therefore, to introduce the neighborhood structure,Storath et al. [55] proposed nonnegative fused LASSO regularization and achieved edge-preserving and noise-reducing reconstruction for MPI. The form of the fused LASSO regularization is as follows:

12 $$\begin{aligned} \underset {\mathbf {c}\ge 0}{\arg \min } \frac{1}{2}\Vert \mathbf {Sc-u}\Vert _2^2+\alpha TV(\mathbf {c})+\beta \Vert \mathbf {c}\Vert _1 \end{aligned}$$

where $TV(\mathbf {c})$ denotes the total variation that promotes a sparse edge. $\Vert \mathbf {c}\Vert _1$ represents the $L_1$ norm. $\alpha$ and $\beta$ are regularization parameters. Storath et al. [55] also introduced nonnegativity to model a (12) and defined it as nonnegative fused LASSO regularization. Compared with Tikhonov regularization, nonnegative fused LASSO regularization is more robust to Gaussian noise. In addition, the reconstructed images showed sharp boundaries and uniformity. Regularization technology plays an important role in the field of SM-based MPI reconstruction and is an important approach to further improve image quality.

## X-space methods for MPI reconstruction

X-space methods are another important research topic for MPI reconstruction [39]. X-space and SM-based reconstruction differ significantly. Specifically, x-space can achieve a real-time reconstruction by dividing the velocity of the FFR based on the linear shift-invariant (LSI) system.

Mathematically, the theory of one-dimensional (1D) x-space in MPI can be described as follows:

13 $$\begin{aligned} u(t)=\mathbf {p(r)}G\dot{\mathbf {r}}_s(t)c(\mathbf {r})*h(\mathbf {r}) \end{aligned}$$

where $\dot{\mathbf {r}}_s(t)$ denotes the velocity of the FFR (In this review, we consider FFP as an example.). $\mathbf {p(r)}$ is the sensitivity of the receiver coil, and G denotes the gradient strength. $h(r)=\frac{d\mathbf {M}}{d\mathbf {H}}$ represents the point spread function (PSF), which is the derivative of the Langevin function:

14 $$\begin{aligned} h(\mathbf {r})=m\dot{\mathcal {L}}\left( \xi \mathbf {H}\right) \end{aligned}$$

The raw MPI image was obtained using a simple two-step velocity-compensation process. MPI reconstruction is performed by gridding the induced voltage signal to the velocity of the FFP:

15 $$\begin{aligned} \hat{c}(\mathbf {r})=c(\mathbf {r})*h(\mathbf {r})=\frac{u(t)}{\mathbf {p(r)}G\dot{\mathbf {r}}_s(t)} \end{aligned}$$

where $\hat{c}(\mathbf {r})$ denotes the raw MPI image, which can be described as the convolution of the real MPI image $c(\mathbf {r})$ with a PSF.

The MPI voltage signal can be regarded as a sampling operation in the raw MPI image $\hat{c}(\mathbf {r})$ at the instantaneous position of the FFP. Therefore, $\hat{c}(\mathbf {r})$ can be reconstructed by the velocity compensation and meshing of the MPI voltage signal based on the position of the FFP.

In 2011, Goodwill and Conolly [56] extended 1D x-space theory to 2D and 3D x-space reconstruction. They proved that 3D MPI is also an LSI imaging process and derived the 3D PSF of MPI. Furthermore, they verified the feasibility of the 3D x-space method on a theoretical basis and conducted 2D MPI verification based on an x-space MPI scanner. However, the quality of reconstructed images must be improved. A year later, Goodwill et al. [57] proposed the projection x-space MPI theory based on FFL scanning. They used permanent magnets to generate the FFL for the first time and provided the first FFL-based MPI images. Fast 3D MPI scans were performed using a FFL-based projection x-space to further improve image quality and sharpness. However, classical x-space methods ignore the relaxation effects of SPIOs and assume SPIOs are instantaneously magnetized with the magnetic field, possibly diminishing the MPI signal and causing artifacts in the reconstructed images [58, 59].

### X-space methods based on relaxation models

Classical x-space methods use the Langevin model under the adiabatic hypothesis to describe the magnetization process of SPIOs:

16 $$\begin{aligned} \mathbf {M}_{L}(\mathbf {r},t)=m\mathcal {L}(\xi \mathbf {H}) \end{aligned}$$

**Fig. 5:**
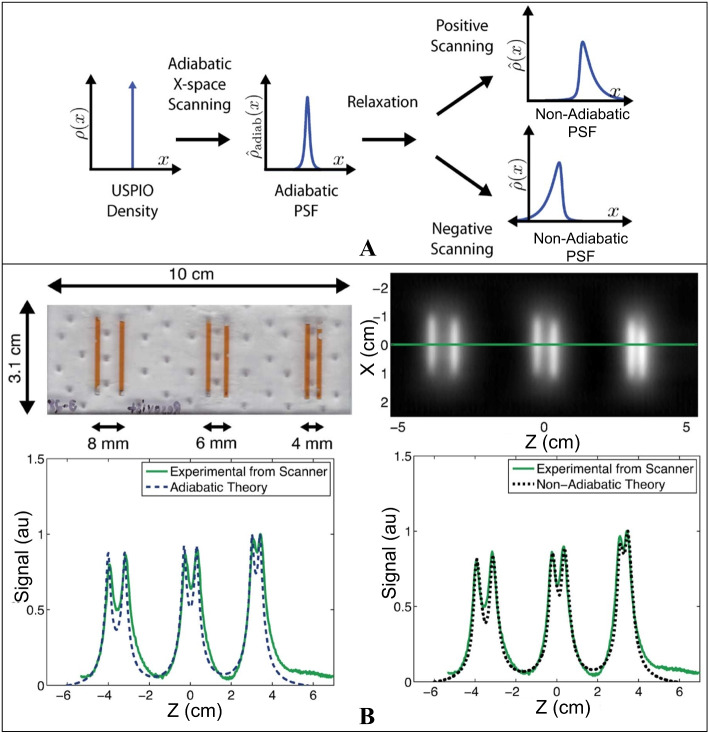
**A** Comparison between adiabatic and nonadiabatic x-space scanning. The blurs caused by the relaxation effects occur in two scanning directions, which lead to nonidentical PSFs. **B** Experiments and reconstructed results of line phantoms with different spacing. For more images see ref. [60]

However, in practice, SPIOs cannot operate in an ideal adiabatic environment. In a nonadiabatic environment, SPIOs change with the magnetization delay, which is often referred to as relaxation effects or relaxation time. Therefore, errors occur when using the Langevin model to describe the magnetization process of SPIOs under adiabatic assumptions, leading to blurred and inaccurate x-space reconstructed images (Fig. 5 A).

Several research groups have proposed improved x-space methods based on relaxation models to account for the relaxation effects and accurately describe the magnetization process. Croft et al. [60] theoretically and experimentally demonstrated the blurring of x-space images and deterioration of resolution and SNR caused by relaxation effects. Furthermore, they proposed a simple relaxation model called the Debye model to achieve a desirable signal strength and resolution (Fig. 5 B). A differential equation in the Debye model is as follows:

17 $$\begin{aligned} \frac{d\mathbf {M}_{D}(\mathbf {r},t)}{dt}=-\frac{\mathbf {M}_{D}(\mathbf {r},t)-\mathbf {M}_{L}(\mathbf {r},t)}{\tau } \end{aligned}$$

where $\mathbf {M}_D(\mathbf {r},t)$ denotes the Debye model that describes nonadiabatic magnetization. $\tau$ denotes the relaxation-time constant. Furthermore, by solving this differential equation, the following temporal convolution formulation can be obtained:

18 $$\begin{aligned} \mathbf {M}_D(\mathbf {r},t)=\mathbf {M}_{L}(\mathbf {r},t)*\frac{1}{\tau }\text {exp}(-t/\tau )p(t)=\mathbf {M}_{L}(\mathbf {r},t)*q(t) \end{aligned}$$

where *p*(*t*) denotes the Heaviside function, with $\tau >0$. Nonadiabatic magnetization can be described as the temporal convolution of the adiabatic magnetization and convolution kernel *q*(*t*) (*q*(*t*) denotes the relaxation process). Correspondingly, the nonadiabatic raw image is described as the temporal convolution of the adiabatic raw image and convolution kernel:

19 $$\begin{aligned} \hat{c}_D(\mathbf {r})=\hat{c}_{L}(\mathbf {r})*q(t)=\left( c(\mathbf {r})*h(\mathbf {r}) \right) *q(t) \end{aligned}$$

$\hat{c}_{L}$ denotes a raw image based on the Langevin model (ideal adiabatic conditions). The nonadiabatic X-space method based on the Debye relaxation model showed excellent consistency with the measured signals from the Berkeley MPI scanner [60].

The relaxation effect is caused by Néel and Brownian rotations of the SPIOs. In MPI, two rotations are coupled, and both rotations have their own relaxation times. However, the Debye model uses a relaxation time constant $\tau$ to represent all rotations, which cannot describe the dynamic magnetization process or rotation mechanism in a complete and accurate manner [61]. Therefore, in recent years, many theoretical models have been developed to accurately describe the relaxation effects of SPIOs. Löwa et al. [62] proposed that the magnetization behavior of SPIOs should be quantified as a function of the excitation magnetic field, properties of SPIOs, and surrounding environment. At present, the Landau-Lifshitz-Gilbert model is the most comprehensive model for exploring the relaxation effect [63]. However, an obvious error is observed between the simulation and measurement results, and significant computational power is required to generate the solution [61]. The Fokker-Plank model explores the dependence of the relaxation time on the excitation field [64]. However, the solutions did not couple the Néel and Brownian rotations [65, 66]. The complex relaxation effect has an important influence on the reconstruction results of the x-space; therefore, conducting relevant research is crucial.

## AI methods for MPI reconstruction

The development of deep learning technology based on AI has inspired new approaches to MPI reconstruction. Currently, AI is primarily applied in MPI reconstruction [67, 68], SM recovery [69], and image postprocessing [70].

**Fig. 6:**
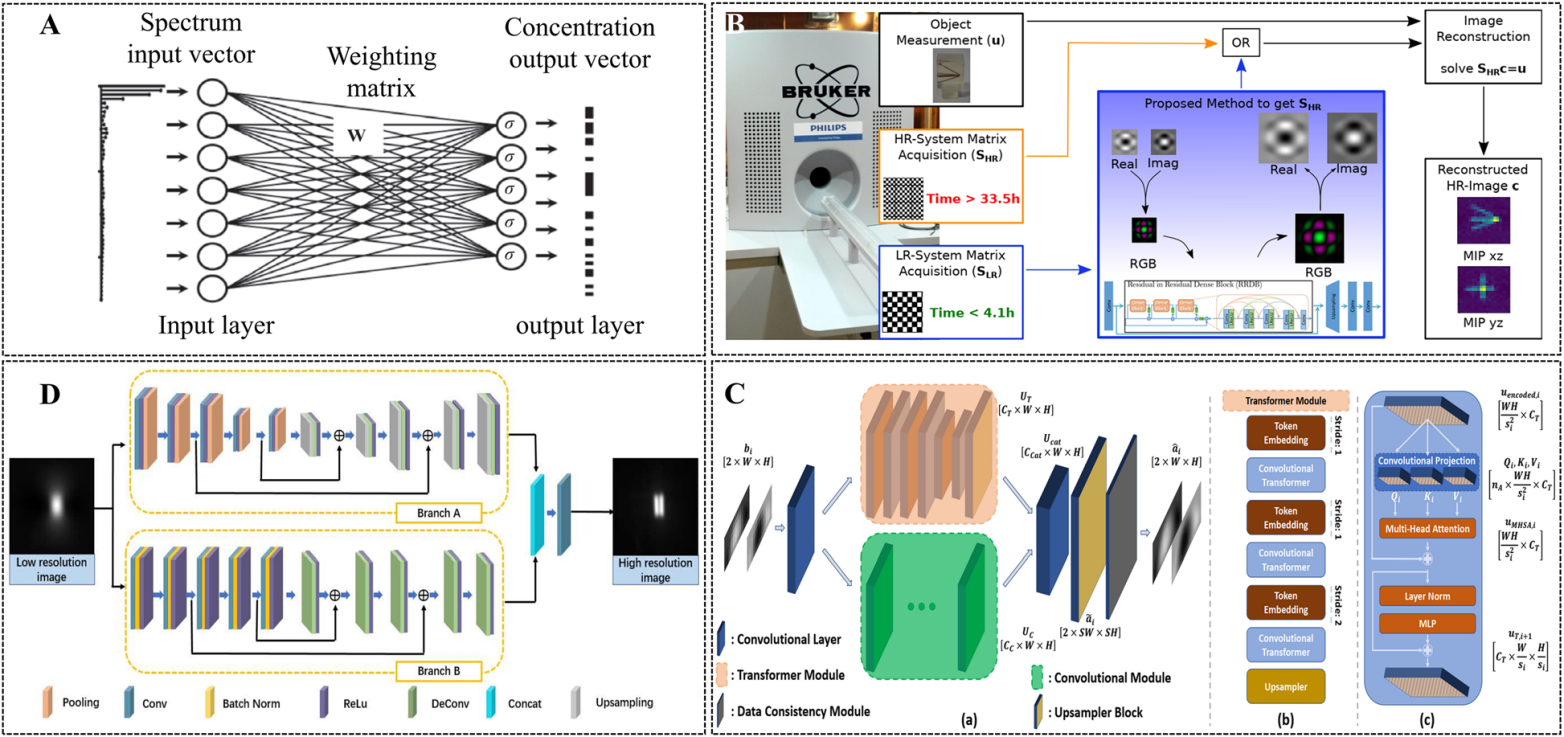
Deep learning networks used in MPI. **A** Architecture of a single-layer neural network [67]. **B** Overview of the data flow of 3d-SMRnet[69]. **C** Schematic of TranSMS [73]. **D** Main framework of FDS-MPI [70]

In 2017, Chae [67] proposed the reconstruction of MPI images using a single-layer fully connected network (Fig. 6 A). The input of the network is the voltage $\mathbf {u}$ in the frequency domain, and the output is the concentration distribution of the SPIOs $\mathbf {c}$: $\mathbf {c}=\delta (\mathbf {W}\mathbf {u})$. $\delta$ is the activation function, and $\mathbf {W}$ represents the weighting matrix. The author constructed 30000 training datasets and 1000 test sets using 1D simulation. The test images were all reconstructed well by the trained network. The results showed that a neural network structure is expected to be a better tool for MPI image reconstruction because of its capacity to overcome the low incoherence of the inverse kernel through the classification property. Subsequently, Dittmer et al. [68] proposed a novel reconstruction framework based on a deep image prior (DIP). The basic idea of DIP is to use an untrained neural network that implicitly encodes a priori to perform a reliable reconstruction [71]. Compared with traditional variational and iterative regularization, it has significant advantages in terms of image quality [72].

The SM recovery problem was transformed into a super-resolution reconstruction problem for deep learning approaches. Baltruschat et al. [69] proposed the use of a 3D-SM recovery network (3D-SMRnet) to recover a 3D SM with an undersampling rate of less than 1.6% (Fig. 6 B). Their results showed that 3D-SMRnet is superior to the CS method in terms of SM recovery quality, reconstructed image quality, and performing time. In addition, Güngör et al. [73] proposed an SM super-resolution method based on transformer network architecture (TranSMS) (Fig. 6 C). Compared with state-of-the-art CS and deep learning baselines, TranSMS enables low-error SM recovery and high-quality MPI image reconstruction.

The advantage of deep learning technology in the field of image processing also inspired MPI image postprocessing. Shang et al. [70] designed an end-to-end dual-sampling convolutional neural network to improve the spatial resolution of MPI (FDS-MPI) (Fig. 6 D). The results of the simulation, phantom, and in vivo experiments demonstrated the advantages of FDS-MPI in improving MPI image resolution. Deep learning technology plays an important role in signal and image processing. It has been applied to many medical modalities such as CT, MRI, and ultrasound. This will definitely shine in benefit MPI in the future.

## Current open sources for MPI

Many countries, such as Germany, United States, Turkey, China, Japan, and South Korea, have conducted research on MPI. More importantly, many groups have created open sources that allow novice groups to conduct MPI-related research as quickly as possible. In this section, we summarize widely used open sources.

**Fig. 7:**
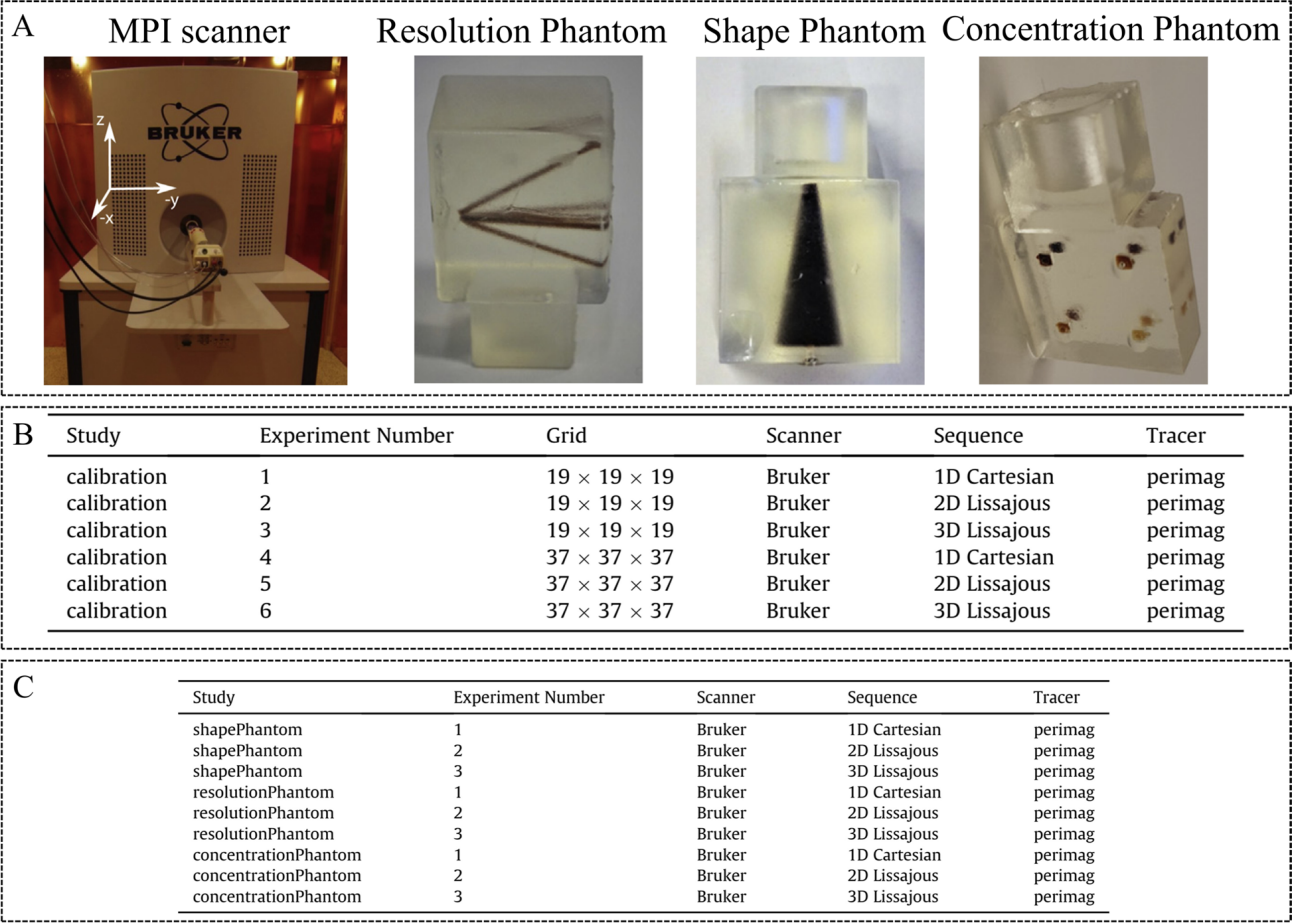
Open MPI data [72]. **A** MPI scanner and phantoms used for measurements. **B** Calibration datasets. **C** Phantom datasets

Knopp et al. [72] published an article about Open MPI data in 2020, which provides experimental MPI and related data processing code for free. This benefits research groups without MPI systems to understand and analyze MPI experimental data. This dataset is stored in the MDF format, which is an open-document standard format. A pre-clinical MPI scanner (Bruker, Ettlingen) was used to acquire the MPI data, including three different phantoms and three different imaging sequences, as shown in Fig. 7. The CAD data of the phantoms are also provided. Furthermore, the Open MPI data also contain the SM based on the calibrations for different mesh densities (Fig. 7 B). Several groups have conducted research on image reconstruction, SM recovery, and other aspects based on Open MPI data [44, 68, 69].

In addition, Tian’s team from the Chinese Academy of Sciences has conducted several MPI studies. They set up an MPI website (MPILabs, http://mpilab.net/en/simulation) that exhibits relevant academic research, open-source experimental data, an algorithm framework, and a software platform. Notably, the software platform MPIRF developed in Python 3.8 integrates SM-based and x-space algorithms to realize MPI reconstruction from the voltage signal of SPIOs to the image [74].

Another public MPI site, open-Source MPI (https://os-mpi.github.io), is offered by Harvard Medical School and Massachusetts Institute of Technology. It publishes the MPI system design and basic components, as well as the associated auxiliary simulation algorithms. These MPI open sources have greatly promoted the development of MPI worldwide.

## Conclusions and outlook

As a novel imaging technology, MPI has wide research and application prospects. This study primarily reviews MPI reconstruction techniques, the SM-based and x-space methods. For these, we provide a detailed review of the SM acquisition and reconstruction methods based on a regularization strategy. In addition, the x-space reconstruction theory and corresponding improved algorithms based on the relaxation model were described. We also reviewed recent deep learning applications in MPI reconstruction and current open sources of MPI.

This review demonstrates the multifaceted nature of MPI. The main challenge for SM-based reconstruction in the future may involve obtaining the SM more quickly and accurately through modeling, measurement, or simulation; this requires a detailed understanding of the SM. In addition, the introduction of prior knowledge and deep learning technology to further improve the reconstruction performance of SM-based reconstruction remains a research focus. In terms of x-space methods, establishing a more accurate magnetization model to describe the relaxation or hysteresis effect is an important challenge to overcome. Furthermore, multi-contrast or multi-color reconstruction based on the specific physical properties of SPIOs, as well as sequence development and corresponding image reconstruction based on novel hardware design, are important challenges and interesting hot spots.

In the past 20 years, because of the tireless efforts of scientists worldwide, MPI has progressed considerably in equipment development, magnetic particle preparation, and reconstruction algorithm research, which have further promoted the application of MPI in clinical settings. We expect this review to provide references for MPI researchers and promote the development and future clinical applications of MPI.

## Data Availability

All data generated or analyzed in this study are included in the published article [and its supplementary information files].

## Abbreviations

MPI: Magnetic particle imaging
SPIO: Superparamagnetic iron oxide particle
FFR: Field-free region
FOV: Field of view
CT: Computed tomography
MRI: Magnetic resonance imaging
PET: Positron emission tomography
SPECT: Single-photon emission computed tomography
FFL: Field-free line
FFP: Field-free point
3D: Three-dimensional
SM: System matrix
CS: Compressed sensing
SNR: Signal-noise ratio
SVD: Singular value decomposition
LSI: Linear shift-invariant
PVT: Paraventricular thalamic nucleus
1D: One-dimensional
2D: Two-dimensional
AI: Artificial intelligence
DIP: Deep image prior
3D-SMRnet: 3D-SM recovery network
